# The Effects of Digital Health Interventions for Pulmonary Rehabilitation in People with COPD: A Systematic Review of Randomized Controlled Trials

**DOI:** 10.3390/medicina60060963

**Published:** 2024-06-11

**Authors:** Aseel Aburub, Mohammad Z. Darabseh, Rahaf Badran, Owis Eilayyan, Ala’a M. Shurrab, Hans Degens

**Affiliations:** 1 Department of Physiotherapy, Applied Science Private University, Amman 11931, Jordan; a_abualrob@asu.edu.jo; 2 Department of Physiotherapy, School of Rehabilitation Sciences, The University of Jordan, Amman 11942, Jordan; 3 Department of Physiotherapy, Faculty of Applied Medical Sciences, Middle East University, Amman 11831, Jordan; 4 Department of Physical Therapy, Al-Ahliyya Amman University, Amman 19111, Jordan; o.eilayyan@ammanu.edu.jo; 5 Department of Basic Medical Science, Faculty of Medicine, Al-Balqa Applied University, Al Salt 19117, Jordan; alaa.shurrab@bau.edu.jo; 6 Department of Life Sciences, Institute of Sport, Manchester Metropolitan University, Manchester M1 5GD, UK; h.degens@mmu.ac.uk; 7 Institute of Sport Science and Innovations, Lithuanian Sports University, LT-44221 Kaunas, Lithuania

**Keywords:** pulmonary rehabilitation, digital health intervention, COPD, physiotherapy, exercise capacity

## Abstract

*Background and Objectives*: Chronic Obstructive Pulmonary Disease (COPD) is the third most common cause of death globally. Pulmonary rehabilitation (PR) programmes are important to reduce COPD symptoms and improve the quality of life of people with COPD. Digital health interventions have recently been adopted in PR programmes, which allow people with COPD to participate in such programmes with low barriers. The aim of this study is to review and discuss the reported effects of digital health interventions on PR outcomes in people with COPD. *Materials and Methods:* To achieve the study goals, a systematic literature search was conducted using PubMed (MEDLINE), CINAHL, AMED, SPORTDiscus and the Physiotherapy Evidence Database. Randomised clinical trials (RCTs) were included if they met specified criteria. Two reviewers independently checked titles, abstracts, and performed full-text screening and data extraction. The quality assessment and risk of bias were performed in accordance with the PEDRO scale and Cochrane Risk of Bias tool 2, respectively. *Results*: Thirteen RCTs were included in this systematic review with 1525 participants with COPD. This systematic review showed the potential positive effect of digital health PR on the exercise capacity—measured by 6- and 12-min walking tests, pulmonary function, dyspnoea and health-related quality of life. There was no evidence for advantages of digital health PR in the improvement of anxiety, depression, and self-efficacy. *Conclusions*: Digital health PR is more effective than traditional PR in improving the pulmonary and physical outcomes for people with COPD, but there was no difference between the two PR programmes in improving the psychosocial outcomes. The certainty of the findings of this review is affected by the small number of included studies.

## 1. Introduction

Pulmonary rehabilitation (PR) is defined, according to the official American Thoracic Society/European Respiratory Society statement, as a “comprehensive intervention based on a thorough patient assessment followed by patient-tailored therapies that include, but are not limited to exercise training, education, and behaviour change, designed to improve the physical and psychological condition of people with chronic respiratory disease and to promote long-term adherence to health-enhancing behaviours” [1]. Chronic respiratory diseases include, but are not limited to, chronic obstructive pulmonary disease (COPD), which is a progressive lung condition characterized by airway inflammation, obstructed airflow, and a reduction in lung function leading, ultimately, to breathing difficulty [2].

COPD was the third most common cause of death worldwide in 2019 [3]. COPD may cause anxiety, depression, peripheral muscle fatigue, dyspnoea, reduction in the activities of daily living, and an increased incidence of hospitalisation. This has a negative effect on the quality of life of an individual and imposes a burden on healthcare systems worldwide [4,5]. PR is the gate to reduce COPD symptoms, and to improve exercise capacity and overall quality of life [6]. However, COPD patients face difficulties in accessing traditional PR services, whether due to high costs, limited infrastructure, lack of interest, and, even if they start PR, there is generally a poor adherence rate to PR programmes [6,7,8]. A recent prospective multicentre cohort study found that only 1% of patients hospitalised with COPD exacerbation utilised PR after discharge, with no in-hospital interventions associated with increased PR use [9]. This highlights the significant underutilisation of PR among COPD patients and the urgent need for effective interventions to increase PR uptake. There is thus a significant need to overcome these barriers, and with the technological revolution in recent years, the innovation of digital health interventions has triggered considerable interest in developing healthcare delivery, including PR for COPD patients. Digital technology is considered safe and feasible, and encompasses a wide range of technologies. Digital health is defined as technology employed to deliver remote care beyond the use of a telephone (e.g., the delivery of care using the internet, virtual reality systems, wearable devices and mobile apps) [10,11], which may provide a platform to increase the accessibility, flexibility, and effectiveness of PR programmes. It should be mentioned that digital health encompasses terms such as telehealth, telemedicine, eHealth, etc.

Despite the growing interest in digital health interventions to assist PR programmes, no systematic reviews have been conducted to explore the effectiveness of such interventions on people with COPD. Therefore, in this review, we will explore the effectiveness of using digital health interventions as PR interventions for people with COPD, highlighting the potential benefits and implications for clinical practice.

## 2. Materials and Methods

### 2.1. Objective

The aim of this study was to review and discuss the reported effects of digital health interventions on respiratory rehabilitation outcomes in people with COPD.

### 2.2. Design

A systematic review with narrative synthesis and quality assessment of published literature was conducted. In this systematic review, we considered the definition of “digital health” as a technology employed to deliver remote care beyond the use of a telephone (e.g., the delivery of care using the internet, virtual reality systems, wearable devices and mobile apps) [10,11].

### 2.3. Study Protocol

The protocol of the systematic review is registered in the international prospective register of systematic reviews database (PROSPERO) (PROSPERO 2023, CRD42023475514). To not limit the search, outcome measures were not limited to any keywords.

### 2.4. Search Strategy

The search was conducted through EBSCO using the following databases: PubMed (MEDLINE), CINAHL, AMED, SPORTDiscus and Physiotherapy Evidence Database (PEDro). These databases were selected because of their comprehensive coverage of articles related to digital health, pulmonary rehabilitation in COPD, physiotherapy and exercise-related research. The search was conducted for papers published between 1 January 1970 and 31 December 2023. All search records were managed using Endnote 21 (Clarivate Analytics, Philadelphia, PA, USA). To assure the reproducibility and accuracy of the search, the Medical Subject Headings (MeSH) were used.

To facilitate replication of findings, customized inquiries were performed for each database in accordance with the PRISMA guidelines. To gain a more comprehensive understanding of current research, grey literature was surveyed via the World Health Organization (WHO) International Clinical Trials Registry platform.

A summary of the keywords and search strategy is presented in Table 1. The rationale for selecting these specific databases and search terms was to capture a broad spectrum of relevant studies and ensure comprehensive coverage of the topic.

### 2.5. Inclusion and Exclusion Criteria

The inclusion and exclusion criteria were determined using the PICOS framework, which encompasses population, intervention, comparison, outcome measures, and study design. Articles were included if they were randomised controlled trials (RCTs) investigating the effects of digital health for pulmonary rehabilitation in adult men and women, aged >18 years old and diagnosed with COPD. Articles were excluded if they were not RCTs; did not use digital health for pulmonary rehabilitation; were not published in English; or were conference abstracts and any study that included participants with any other neurological, musculoskeletal, or cognitive disorders.

### 2.6. Study Selection

After conducting the search and eliminating duplicate entries, two reviewers (RB and AS) independently assessed the relevance of titles and abstracts. Subsequently, the same reviewers independently evaluated the full texts of relevant trials to determine their eligibility based on the inclusion and exclusion criteria.

### 2.7. Data Extraction and Synthesis

A meta-analysis was not performed due to the heterogeneity of the included trials. Despite the similar designs of the trials, variations in intervention types and outcome measures prevented a meaningful statistical aggregation of results. Therefore, a narrative synthesis approach was employed to qualitatively analyse and interpret the findings.

Information extracted from the included trials was tabulated and included the following: names of the authors, study designs, sample sizes, demographics such as sex and age, digital health services offered (including utilized technology), prescribed exercises, reported outcome measures, and principal findings. Additionally, details regarding the frequency, duration, intensity, and nature of exercises were documented when accessible.

### 2.8. Quality Assessment and Risk of Bias of the Included Trials

The evaluation of the included trials’ quality was conducted using the PEDro scale, a recognized and dependable tool specifically designed for assessing the quality of interventional studies within the field of physiotherapy [12,13]. PEDro scores were not employed as inclusion or exclusion criteria; rather, they served as a foundation for synthesizing the best evidence and identifying the strengths and weaknesses of each study. Scores on the PEDro scale range from 0 to 10, with scores of 9–10 denoting excellent quality, 6–8 indicating good quality, 4–5 reflecting low quality, and scores below 4 suggesting poor quality [14].

The risk of bias in the included trials was evaluated using the Cochrane Risk of Bias tool 2 (CROB 2). Two reviewers (RB and AS) independently assessed the risk of bias. The CROB 2 assessment encompassed the following: (1) bias stemming from randomization criteria; (2) bias due to deviations from intended interventions; (3) bias arising from missing outcome data; (4) bias in the measurement of outcomes; and (5) bias in the selection of reported results.

## 3. Results

### 3.1. Study Selection

**Figure 1:**
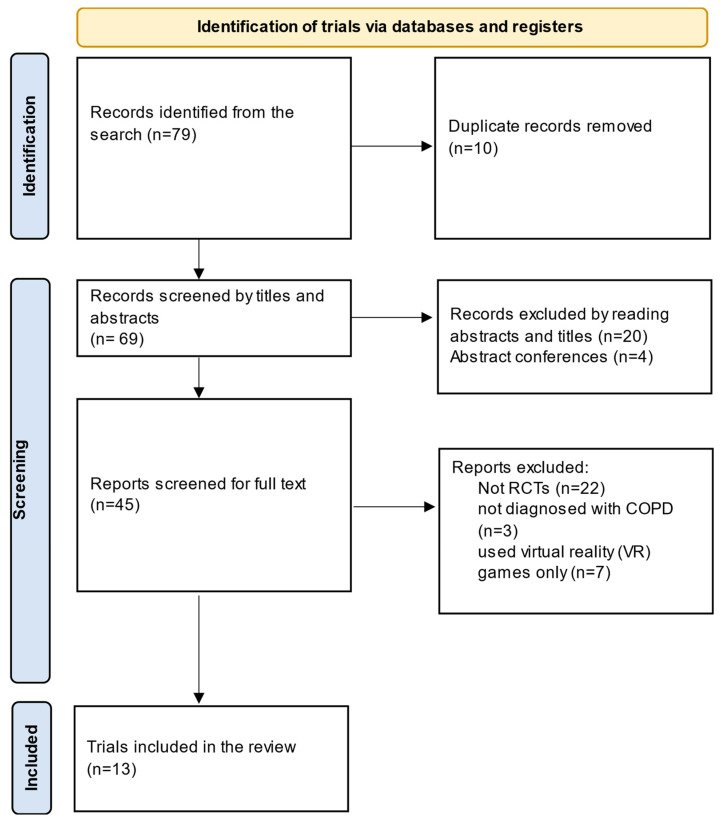
The PRISMA flowchart for the search records and the included trials.

The initial search identified 79 titles, leaving 69 after the removal of duplicates. After screening the titles and abstracts, 24 trials were excluded: 4 were conference abstracts, 20 were not RCTs. After full text screening, it appeared 3 trials included participants who were not diagnosed with COPD, 7 used virtual reality games only and 22 trials were not RCTs. No relevant trials were found in the grey literature. Consequently, 13 trials were included in the review: 12 were RCTs, and 1 was a parallel group noninferiority trial. The included trials recruited 1525 participants with COPD. The Preferred Reporting Items for Systematic Reviews and Meta-Analyses (PRISMA) flowchart indicating the included and excluded trials is represented in Figure 1. The data extracted related to the respiratory function/symptoms of the studies included in this review are presented in Table 2. Other non-respiratory outcome measures are presented in Table S1 as supplementary material. A meta-analysis was not feasible because of the heterogeneity of the included trials.

### 3.2. Study Characteristics and Digital Health Technology Utilized

Two trials used Web-based applications [15,16], five trials used video conferencing [17,18,19,20,21], one trial used online sessions [22], and five trials used smart phone applications [23,24,25,26,27]. The digital technology used in the pulmonary rehabilitation programmes in these trials focused on different types of exercise (endurance, strengthening, aerobic, breathing, flexibility, stretching, and resistance). Five trials used an exercise programme combined with an education session [16,17,20,25,26]. The study characteristics are summarized in Table 2. The majority of these trials were conducted in Europe (*n* = 8) [19,20,21,22,23,24,25,27], one trial in North America [15], one trial in Australia [18], two trials in Asia [16,26] and one trial in Canada [17].

**Table 2:** Summary of included trials that investigated the Effects of Digital Health Interventions for Pulmonary Rehabilitation in People with COPD: A Systematic Review of Randomized Controlled Trials (*n* = 13).

Author (Year)	Location of Trial	Sample Size	Age Years (Mean ± SD)	Technology Utilized	Exercise Prescription	Comparison Group	Key Findings
Nguyen et al., (2008) [15]	USA	eDSMP: 26 fDSMP: 24 COPD: mild to severe	Total: (69.5 ± 8.5) M:F = 22:17 eDSMP: (68.0 ± 8.3) M:F = 11:8 fDSMP: (70.9 ± 8.6) M:F = 11:9	Web-based application	Duration for intervention and follow-up (0, 3, 6 months). F: endurance 4x/wk or resistance 3x/wk. I: Light to moderate Borg scale. T: 30 min/session. T: Endurance, resistance exercise.	Education, skills training, and ongoing support for dyspnoea self-management, including independent exercise.	- CRQ- dyspnoea with ADL improved in both groups from baseline to 3 and to 6 months. - 6MWT ↓) in the fDSMP and ↑ in the eDSMP over time (*p* = 0.05).
Stickland et al., (2011) [17]	Canada	Standard PR: 262 Telehealth PR: 147	Standard PR: (69.5 ± 9.7) M:F = 125:137 Telehealth PR: (69.2 ± 8.6) M:F = 78:69	Video conferencing	Duration for intervention in both groups: 8 weeks, follow-up at 6 months. F: 2x/week for 8 weeks. I: Intensity personalised based on patient symptoms and capacity. T: 2 h/session. Education session for 1 h/session. T: aerobic, resistance, flexibility, breathing exercise.	F: 2x/wk for 8 weeks. I: Personalised based on patient symptoms and capacity. T: 2 h/session. Education 1 h/session. T: aerobic, resistance, flexibility and breathing exercise.	- SGRQ subscale and total scores with PR↑ in both groups (*p* < 0.05) - 12 MWT↑ in both groups (*p* < 0.05) - SGRQ and 12 MWT after 6 months follow-up, no significant differences in both groups
Tabak et al., (2014) [23]	Netherlands	IG: 18 CG: 16 COPD: mild to very severe	IG: (65.2 ± 9.0) M:F = 8:6 CG: (67.9 ± 5.7) M:F = 11:5	Smart-phone application (app)	Both groups received usual care (medication/physiotherapy) for 4 weeks. IG: F: 4 days/week. T,T: Activity couch walking till 22.00 h. CG: usual care.	Usual care (medication/physiotherapy).	- CCQ improved in IG (*p* = 0.046) but not in CG (*p* = 0.89)
Bourne et al., (2017) [24]	UK	Online: 64 Face-to-face: 26 COPD: mild to severe	Online: (69.1 ± 7.9) M:F = 41:23 Face-to-face: (71.4 ± 8.6) M:F = 18:8	PR programme (myPR)	- Duration: 6 weeks both groups. - Online (myPR): F: 2 - 5x/wk. I: Borg score measurement. T: duration of exercise increased by 30 s, starting from 60 s in week 1, to 3½ min in week 6. T: 10 exercises: biceps curls, squats, push ups against wall, leg extensions in sitting position, upright row with weights, sit-to-stand, arm swings with a stick, leg kicks to the side, arm punches with weights, step-ups.	Face to face: F: 2 supervised sessions and 3 at home per week. T: 10 exercises carried out by myPR.	- 6MWT difference between groups for the ITT in favour of IG was 23.8 m. - SRQ and MRC dyspnoea suggested non-inferiority for the online PR.
Tsai et al., (2017) [18]	Australia	TeleG: 20 CG: 17 COPD: mild to severe	TeleG: (73 ± 8) M:F = 12:7 CG: (75 ± 9) M:F = 6:11	Video conferencing	Duration of intervention: 8 weeks for both. TeleG: F: 3/week. I: 60–80% Peak cycle work rate OR 80% of 6MWT speed (walking training). T: Cycle ergometry, walking training and resistance exercise. T: 15–30 min.	CG: Usual medical management including optimal pharmacological intervention.	- ESWT improved more in TG compared to CG (*p* = 0.001). - 6MWT improved more with TG but not significant difference between both groups (*p* = 0.16). - ISWT improved more with TG but not significant difference between both groups (*p* = 0.66). - CRDQ improved more in TG compared to CG (*p* = 0.07).
Vasilopoulou et al., (2017) [19]	Greece	Group A: 50 Group B: 50 Group C: 50 COPD: moderate to very severe	Group A: (66.9 ± 9.6) M:F = 44/3 Group B: (66.7 ± 7.3) M:F = 38/12 Group C: (64.0 ± 8.0) M:F = 37/13	Telephone, video conference, tablet	Duration of intervention and follow-up: 2, 12 months. Group A: F: 144 sessions over 12 months. I: Each patient with a resource executes the exercise (depends in patient status). T: Arm and leg exercise. Group B: Participants visited the hospital 2/wk for 12 months. F: 96 sessions over 12 months.	Group C: optimal pharmacotherapy oxygen therapy, vaccination, regular follow-up.	- Both group A and B lower rate of acute exacerbation and hospitalisations over 12 months of follow-up compared to group C (*p* < 0.001). - Both group A and group B improved 6MWT over 12 months of follow-up (*p* = 0.011) more compared to group C. - Both group A and B improved with SGRQ, CAT and MRC over 12 months of follow-up more compared to group C.
Wang et al., (2017) [16]	China	CG: 68 IG: 62 COPD: moderate to very severe	CG: (71.9 ± 8.1) M:F = 36:29 IG: (69.3 ± 7.8) M:F = 21:34	Web-based coaching programme	All participants received usual care before discharge. Duration: 12 months. EHR: Health education, and pulmonary rehabilitation instructions. Pulmonary rehabilitation: T: Abdominal contraction, lip breathing, respiratory muscle exercise, aerobic exercise.	Usual care before discharge.	- FEV1 improved more at 3 and 12 months compared to CG (*p* < 0.001). - FVC% improved in both groups, (*p* ≤ 0.01). After 12 months follow-up, IG had higher scores compared to CG (*p* = 0.001). - PEF improved with IG (*p* < 0.001) but not with CG (*p* = 0.56). After 12 months follow-up, IG had higher scores compared to CG (*p* < 0.001). - MMEF improved in both IG and CG (*p* ≤ 0.048). - SGRQ improved in both groups (*p* < 0.001). After 12 months follow-up, IG had higher scores (*p* < 0.001) - mMRC improved in both IG and CG (*p* ≤ 0.01). After 12 months, IG had lower scores than CG (*p* < 0.001). - 6MWT after 12 months follow-up, improved more in IG compared to CG (*p* < 0.001).
Godtfredsen et al., (2020) [22]	Denmark	PR: 67 PTR: 67 Stage of COPD: severe	(68.3 ± 9.0) 55% women	Online	Duration of intervention and follow-up: 10 weeks; 3 and 12 months follow-up. PTR Group: F: 10 weeks supervised on-line PTR with a screen at homes.	PR Group: F: 10 weeks of conventional PR at the local site. Both groups performed: T: Endurance, resistance, breathing techniques, nutritional support. T: 10 weeks.	- 6MWD no difference between or within the groups after 12 months of follow-up.
Hansen et al., (2020) [20]	Denmark	PTR: 67 PR: 67 COPD: Moderate to severe	Total: (68.3 ± 9.0) M:F = 60:74 PTR: (68.4 ± 8.7) M:F = 32:35 PR: (68.2 ± 9.4) M:F = 28:39	Video-conference software system	Duration of intervention: 10 weeks. Follow-up: 22 weeks. PTR: F: 3/week for 10 weeks. T: 35 min. T: Warm-up, high repetitive time-based muscle endurance training, and patient education session.	CPR: F: 2/week (1 hospital, for 10 weeks). T: 60 min. T: Warm-up, endurance, resistance training, cool-down, patient education.	- 6MWT improvement not significantly different between groups. - CAT score improved by PTR (*p* = 0.04) compared to PR.
Galdiz et al., (2021) [25]	Spain	CG: 48 IG: 46 COPD: moderate to very severe	CG: (63.0 ± 6.6) M:F = 68.8:31.2 IG: (62.3 ± 8.2) M:F = 65.2:34.8	Mobile phone app	Duration of intervention: 8 weeks. Follow-up: 3, 9, 12 months. Intensive PR: F: 3/week. T: 1 h. T: Weightlifting, leg cycle ergometry, educational sessions.	CG: Usual care. F: Every day. T: At least 1 h. T: Walking, general educational material.	- 6MWT not significantly improved in either group (*p* = 0.188).
Bahadori et al., (2023) [26]	Iran	IG: 38 CG: 38	IG: (44.1 ± 14.1) F:M = 25:10 CG: (47.7 ± 13.8) F:M = 29:6	Android application	Duration of intervention: 6 weeks. IG: F: all day/6 weeks. T: PR education. CG: F: 2/wk. T: PR education. T: 30–60 min.	CG: wore the activity tracker every day and used a smartphone for the assessments but no access to the app.	- CB score after PR education lower in IG (*p* < 0.001) compared to CG (*p* = 0.573).
Spielmanns et al., (2023) [27]	Switzerland	IG: 33 CG: 34 COPD: moderate to very severe	Total: (64.3 ± 7.7) M:F = 34:33 IG: (66.1 ± 6.8) M:F = 17:16 CG: (62.7 ± 8.2) M:F = 17:17	Smart phone application	Duration of Intervention: 3 months. Follow-up: 6 months. IG: F: Daily. I: Progressive increase based on patient feedback. T: 15–20 min. T: Warm up, strength and mobility, stretching exercises.		- CAT score ↓ in IG and ↑ in CG after 6 months of follow-up (*p* = 0.02). - CRQ significant difference with domains of dyspnoea (*p* = 0.033) and fatigue (*p* = 0.028) and not significant with emotional function (*p* = 0.283), mastery (*p* = 0.131), and total score (*p* = 0.056) in both groups after 6 months of follow-up.
Zanaboni et al., (2023) [21]	Norway	TeleRG: 40 UC: 40 CG: 40 COPD: moderate to very severe	TeleRG: (64.9 ± 6 7.1) M:F = 23:17 UC: (64.0 ± 7.7) M:F = 20:20 CG: (63.5 ± 8.0) M:F = 23:17	Video conferencing sessions	Duration of intervention: 6 months. Follow-up: 1 and 2 y. TeleRG: F: 3–5/wk. I: Moderate to high intensity Borg scale. T: Treadmill and resistance exercise. T: At least 30 min. UG: T: Treadmill exercises only as prescribed for Tele-PR group.	CG: Standard care.	- CAT, mMRC improved with TeleRG (*p* = 0.037), compared to CG after 6 months of follow-up. - 6MWD improved in TeleRG and ↓ in CG. - CAT and mMRC improved in UG (*p* = 0.002), (*p* = 0.027) compared to CG after 6 months of follow-up.

All the included trials were randomized controlled trials: eDSMP: Internet-based Dyspnoea Self-management Programme; fDSMP: Face-to-Face Dyspnoea Self-Management Programme; 6MWT: 6 Minute Walk Test; CRQ: Chronic Respiratory Questionnaire; ADL: Activities of Daily Living; PR: Pulmonary Rehabilitation; SGRQ: St George’s Respiratory Questionnaire; 12 MWT: 12 Minute Walk Test; ESWT: Endurance Shuttle Walk Test; MRC: Medical Research Council Dyspnoea Scale; IG: Intervention Group; CG: Control Group; CCQ: Clinical COPD Questionnaire; 6MWD: 6 Minute Walk Distance; ISWT: Incremental Shuttle Walk Test; FEV1: Forced Expiratory Volume in the first second; FVC%: Percent of Forced Vital Capacity; PEF: Peak Expiratory Flow; MMEF: Maximal Mid Expiratory Flow; mMRC: modified Medical Research Council; PTR: Pulmonary Tele-Rehabilitation; UC: Unsupervised Group; ↑, ↓: better or poorer, respectively, improvement compared to control intervention.

### 3.3. Quality Assessment and Risk of Bias

**Figure 2:**
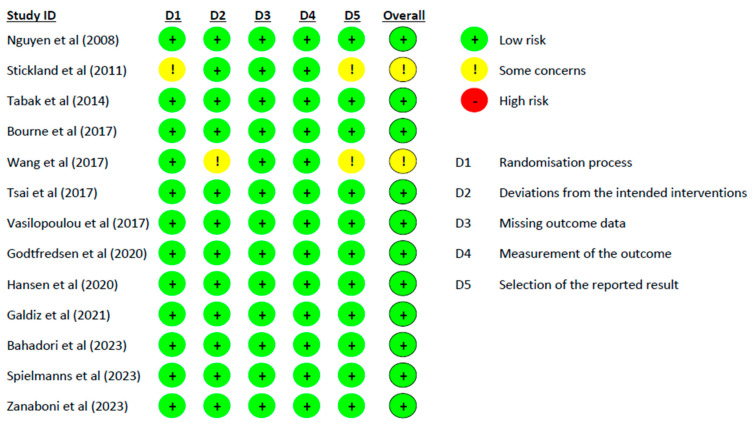
Results of the Cochrane Risk of Bias (CROB2) for the included trials. References cited in the figure are the following: [15,16,17,18,19,20,21,22,23,24,25,26,27].

The quality assessment and risk of bias of the trials were independently assessed by two assessors (RB and AS) using PEDro scores and the CROB2 tool. The PEDro scores of the included trials ranged from 6 to 9, indicating good to excellent quality trials (Table 3). One trial scored 6 because of the bias in blinding of all subjects and random allocation to groups [17], eight trials scored 7 because of bias in blinding of all subjects and allocation concealed [15,16,19,21,22,23,25,26], three trials scored 8 because of bias in blinding of all subjects [18,24,27], and one trial scored 9 because of bias in blinding of therapists and assessors [20]. The risk of bias using the CROB2 tool showed that 11 trials were at a low risk of bias (across all domains) [15,18,19,20,21,22,23,24,25,26,27] and two trials were at “some concern” of risk of bias (some concerns in two domains) [16,17]. A summary of the CROB2 results is shown in Figure 2.

### 3.4. Outcomes Assessed by Digital Health Intervention Trials

The included trials assessed different health outcomes that were categorised into pulmonary rehabilitation outcomes, quality of life, COPD symptoms, lung function, acute exacerbations and hospitalisations, and emergency department visits.

### 3.5. Pulmonary Rehabilitation Outcomes

#### 3.5.1. Exercise Capacity

Thirteen trials used different exercise capacity tests as a primary outcome measure including a 6-min walk test (6MWT), a 12-min walk test (12MWT), an endurance shuttle walking test (ESWT), number of steps per day (PAL), timed up-and-go (TUG), and a 1-min sit-to-stand (1-min STS) (Table 2).

Eight trials used the 6MWT [15,16,19,20,21,22,24,25]: three of them reported a significantly higher improvement in 6MWT results in the intervention group (IG) compared with the control group (CG) (tele-PR vs. regular PR or usual care) [15,16,19]. The other five trials did not report significant differences in improvement between both groups (tele-PR vs. regular PR or usual care) [20,21,22,24,25].

One trial used the 12MWT as an outcome measure for exercise capacity [17] and reported significant improvement in the 12MWT in both the intervention and control groups (tele-PR vs. regular PR). One trial used the ESWT [18] and reported a higher improvement in the intervention group compared with the control group (tele-PR vs. usual care). One trial used the PAL [23] and showed that both groups improved in PAL but no significant differences were found between them (tele-PR vs. usual care). One trial used STS [27]; the test showed that both groups improved but without significant differences between them (tele-PR vs. usual care). One trial used the incremental shuttle walk test (ISWT) [18], where no significant differences in improvement were seen between the two groups (tele-PR vs. usual care) (Table 2) [18].

##### Functional Performance Inventory–Short Form (FBI-SF)

One trial used FBI-SF to assess physical performance in six domains, including body care, household activities, physical exercise, recreation, spiritual activities and social interactions. No significant differences were observed in either group (tele-PR vs. usual care) after the follow-up [18] (Table S1: Supplementary Material).

#### 3.5.2. Quality of Life

##### Chronic Respiratory Disease Questionnaire (CRQ)

Four trials used the CRQ [15,18,25,27]. Nguyen et al. reported an improvement in the activity of daily living (ADLs) in both groups (tele-PR vs. regular PR) at follow-up [15]. Spielmanns et al. presented a significant improvement in both groups (tele-PR and usual care) for dyspnoea and fatigue but not the emotional aspect [27]. However, Galdiz et al. reported no significant differences in either group (tele-PR and usual care) [25] but the emotional aspect only improved in the intervention group. Tsai et al. reported a significant improvement in CRQ total score following training in the intervention group but not in the control group (usual care) (Table S1: Supplementary Material [18].

##### Health-Related Quality of Life (HRQoL)

Two trials used the HRQoL [15,25]. Nguyen et al. reported a significant improvement in both groups (tele-PR and regular PR) after the intervention [15]. Galdiz et al., however, reported a significant improvement in the intervention group (tele-PR vs. usual care) only at follow-up (Table S1: Supplementary Material) [25].

##### The 36-Item Short Form Health Survey (SF-36)

Two trials used the SF-36 [15,25]. Both trials showed a significantly larger improvement in health status in the intervention group compared to the control group (regular PR or usual care) (Table S1: Supplementary Material).

##### St George’s Respiratory Questionnaire (SGRQ)

Four trials used the SGRQ [16,17,19,24]. Two trials reported a significant improvement in both groups (tele-PR and regular PR) [17,19], and one trial reported a significant improvement in the intervention group, but not in the control group (usual care) [16]. The fourth trial did not report significant improvements in SGRQ scores in either group (tele-PR or regular PR) (Table 2) [24].

##### COPD Assessment Test (CAT)

Six trials used the CAT [18,19,20,21,24,27]. Four of them reported an improvement only in the intervention group (tele-PR vs. regular PR or usual care) [20,21,24,27]. One trial reported no significant improvement in either group (tele-PR or regular PR) [18], and two trials reported a significant improvement in both groups (tele-PR and regular PR, or usual care) (Table 2) [19,27].

##### Clinical COPD Questionnaire (CCQ)

Two trials used the CCQ [20,23]. Tabak et al. reported improvement only in the intervention group (tele-PR vs. usual care) [23], whereas Hansen et al. reported no significant differences in improvement between groups (tele-PR vs. regular PR) (Table 2) [20].

##### Pulmonary Rehabilitation Adapted Index of Self Efficacy (PRAISE)

Tsai et al. reported no significant differences in improvement in the PRAISE scores between both groups (tele-PR vs. usual care) at follow-up [18].

##### EuroQol 5-Dimension Questionnaire (EQ-5D)

Hansen et al. (2020) mentioned a significant improvement with EQ-5D in both groups (tele-PR and regular PR) (Table S1: Supplementary Material) [20].

##### Generalized Self-Efficacy Scale (GSES)

Zanaboni et al. (2026) reported no significant differences in improvement between the intervention and control group (tele-PR vs. usual care) (Table S1: Supplementary Material) [21].

#### 3.5.3. COPD Symptoms

##### Medical Research Council Dyspnoea Scale (MRC)

Three trials used the MRC [16,19,21]. Wang et al. reported a significant improvement in both the intervention and control groups (tele-PR and usual care) [16]. Two trials [19,21] reported an improvement in the intervention group only (tele-PR vs. regular PR).

##### Hospital Anxiety and Depression Scale (HADS)

Five trials used the HADS [18,20,22,24,27]. Two trials reported improvement in the intervention group only (tele-PR vs. regular PR or usual care) [18,24], and three trials reported no significant differences in improvement between the groups at follow-up (tele-PR vs. regular PR or usual care) (Table S1: Supplementary Material) [20,22,27].

#### 3.5.4. Lung Function

Only one trial assessed lung function using spirometry [16]. The trial reported improvement in the intervention group, but no improvement in the control group (usual care) in forced expiratory volume in the first second, forced vital capacity, and peak expiratory flow. The maximum mid-expiratory flow significantly improved in both the intervention group and the control group (Table 2) [16].

#### 3.5.5. Acute Exacerbation and Hospitalisations

One trial showed a significantly larger reduction in the incidence of acute exacerbations and hospitalisations in the intervention than the control group (usual care) [21]. Vasilopoulou et al. (2017) reported a significantly lower rate in both the intervention and control groups (tele-PR and regular PR) [19].

#### 3.5.6. Emergency Department Visits (ED)

One trial reported a significantly lower rate in both the intervention and the control group (tele-PR and regular PR) [19], However, ED had less significance with the intervention group only in another study (tele-PR vs. usual care) (Table S1: Supplementary Material) [21].

## 4. Discussion

To our knowledge, this is the first study that reviewed the reported effects of digital health interventions on pulmonary rehabilitation outcomes in people with COPD. A total of 13 RCTs were included in the systematic review with good to excellent quality; this makes the results trustworthy. The findings of this systematic review showed that the digital health interventions had a positive effect on dyspnoea, lung function, HRQoL and the incidence of hospitalizations and ED visits. This supports the use of digital health interventions for pulmonary rehabilitation outcomes.

The results of this review showed a potentially positive effect of integrating the digital health interventions with pulmonary rehabilitation on exercise capacity. The exercise capacity measures cover a range of tests, including the walking, timed up-and-go, and sit-to-stand tests. An improvement was shown in the walking tests (ESWT, 6 and 12MWT) [15,16,17,18,19]. These trials used educational materials and an interaction between the patient and healthcare provider during the tele-rehabilitation programme that in themselves may improve the exercise capacity performance compared with the traditional interventions, as indicated in a meta-analysis among individuals with chronic illness [28].

The findings of this systematic review also suggest that digital health interventions improve dyspnoea symptoms and lung function [16,19,21]. These three trials applied comprehensive rehabilitation programmes, including self-management, to improve dyspnoea and lung function. The literature supports the effectiveness of self-management on dyspnoea [29,30] and lung function [31] among individuals with COPD, and those trials suggest that such improvements can be further enhanced by incorporating digital health interventions in the rehabilitation programme.

The included trials measured HRQoL with different measures, including generic and disease-specific measures. The findings support the positive effect of digital health interventions, and that using digital health results in significant improvements [16,19,21]. This improvement could be achieved by performing exercises [32,33], receiving educational material about COPD and PR [34] and/or applying a self-management approach [30,31].

Seven studies included in this systematic review compared the benefits of tele-PR and regular PR. While two studies showed that the tele-PR improved exercise capacity more than regular PR [15,19], four studies did not find a significant difference in improvement between the two groups [17,20,22,24]. In terms of QOL, two studies showed an enhanced improvement in tele-PR compared to regular PR [19,26], while one study showed no differences [17]. Furthermore, two studies revealed enhanced improvements in COPD signs and symptoms [19,20], and another two studies showed that the difference in improvement was not significant [15,24]. Hansen et al., 2020 [20] showed a significantly larger reduction in depression and anxiety with tele-PR, while Bourne et al., 2017 indicated no differences with regular PR [24]. Lastly, one study compared regular and tele-PR regarding self-efficacy, and it was significantly improved with tele-PR, but not with regular PR [15]. Overall, these observations indicate that tele-PR does result in similar or even better—but never worse—improvements than regular PR and hence support the use of tele-PR as an alternative to regular PR, as it reduces the cost to patients and the healthcare system.

Lastly, the findings did not support the advantages of digital health interventions in the improvement of anxiety, depression and self-efficacy. Typically, pulmonary rehabilitation programmes focus on physical and breathing exercises [35,36] and the effectiveness of such programmes on psychosocial outcomes is apparently not further improved by the incorporation of digital health interventions.

### 4.1. Clinical Implications

The use of digital health interventions to support pulmonary rehabilitation among individuals with COPD improves pulmonary and physical outcomes. It may also reduce the cost of COPD-related sequalae by lowering the rate of exacerbations, hospitalizations, and ED visits. Therefore, this systematic review suggests that these interventions should be integrated with the usual care of people with COPD to maximize the health outcomes of these people.

Although there is a lack of evidence to show that digital PR is better than face-to-face PR, it never led to a worse outcome. Therefore, a personalized approach should be used, with digital PR serving as an adjunct to regular PR [35,36] and perhaps even as an alternative to regular PR as it reduces the cost to patients and the healthcare system.

Digital health PR can take different forms: web-based, smartphone applications and video conferences. The results of this review showed that the effectiveness of digital health PR does not depend on its form; it relies more on the components of the intervention. Digital health PR should include a self-management programme, home monitoring, and an E-health telephone or platform [37]. To optimize the effect of digital health PR, self-management should include interventions to address physical and psychosocial issues.

### 4.2. Future Research

This systematic review highlights that there is a need to conduct more studies on the use of digital health interventions in pulmonary rehabilitation to strengthen the conclusion about the effectiveness of such interventions and to ensure its inclusion in pulmonary rehabilitation guidelines.

### 4.3. Limitations

This study is not without limitations. For example, the included trials were heterogeneous, and therefore a meta-analysis was not conducted. This limits the estimation of the magnitude of the effectiveness of digital health PR. Other limitations include potential publication bias, the varying quality of the included trials, and the exclusion of non-English-language trials.

## 5. Conclusions

This review supports that digital health PR for people with COPD is effective in improving pulmonary and physical outcomes and has no negative impact on psychosocial outcomes. The small number of included trials that used different forms of digital health PR limits the certainty of the findings of this review.

## Figures and Tables

**Table 1:** Summary of keywords used and search strategy.

Search Strategy	Search Strategy
P (population)	S1 = “chronic obstructive pulmonary disease” OR “COPD” OR “Chronic Obstructive Lung Disease” OR “COAD” OR “Chronic Obstructive Airway Disease” OR “Chronic Obstructive Pulmonary Diseases” OR “Airflow Obstruction, Chronic” OR “Airflow Obstructions, Chronic” OR “Chronic Airflow Obstructions” OR “Chronic Airflow Obstruction” OR “Asthma-Chronic Obstructive Pulmonary Disease Overlap Syndrome” OR “Bronchitis, Chronic” OR “Pulmonary Emphysema”
I (Intervention)	S2 = “digital health” OR “internet” OR “web” OR “Physiotherapy” OR “Physiotherapist” OR “online” OR “telemedicine” OR “telehealth” OR “tech” OR “mobile” OR “home” OR “wearable” OR “ehealth” OR “e health” OR “virtual” OR “digital” OR “remote” OR “smartphone” OR “text messaging” OR “community”
C (comparison)	Interventions that include no pulmonary rehabilitation or digital health
O (outcome measures)	Outcome measures were not limited to any keywords
S (study design)	Randomized controlled trials, controlled clinical trials
Combined final search	S1 AND S2

**Table 3:** Results of the PEDro scale for quality assessment for the included randomised controlled trials.

Author (Year)	1. Eligibility Criteria Were Specified	2. Subjects Were Randomly Allocated to Groups	3. Allocation Was Concealed	4. The Groups Were Similar at Baseline Regarding Prognostic Indicators	5. There Was Blinding of All Subjects	6. There Was Blinding of All Therapists Who Administered the Therapy	7. There Was Blinding of All Assessors Who Measured at Least One Key Outcome	8. Measures of at Least One Key Outcome Were Obtained from More than 85% of the Subjects	9. All Subjects for Whom Outcome Measures Were Available Received the Treatment or Control Condition as Allocated	10. The Results of between-Group Statistical Comparisons Are Reported for at Least One Key Outcome	11. Point Measures and Measures of Variability for at Least One Key Outcome Were Reported	Total PEDro Score
Nguyen et al., (2008) [15]	1	1	0	1	0	0	0	1	1	1	1	7
Stickland et al., (2011) [17]	1	0	0	1	0	0	0	1	1	1	1	6
Tabak et al., (2014) [23]	1	1	0	1	0	0	0	1	1	1	1	7
Bourne et al., (2017) [24]	1	1	0	1	0	1	0	1	1	1	1	8
Wang et al., (2017) [16]	1	1	0	1	0	0	0	1	1	1	1	7
Tsai et al., (2017) [18]	1	1	1	1	0	0	0	1	1	1	1	8
Vasilopoulou et al., (2017) [19]	1	1	0	1	0	0	0	1	1	1	1	7
Godtfredsen et al., (2020) [22]	1	1	1	0	0	0	0	1	1	1	1	7
Hansen et al., (2020) [20]	1	1	1	1	0	1	0	1	1	1	1	9
Galdiz et al., (2021) [25]	1	1	0	1	0	0	0	1	1	1	1	7
Bahadori et al., (2023) [26]	1	1	0	1	0	0	0	1	1	1	1	7
Spielmanns et al., (2023) [27]	1	1	1	1	0	0	0	1	1	1	1	8
Zanaboni et al., (2023) [21]	1	1	0	1	0	0	0	1	1	1	1	7

## Data Availability

The data that support the findings of this study are available within the manuscript.

## Supplementary Materials

The following supporting information can be downloaded at: https://www.mdpi.com/article/10.3390/medicina60060963/s1, Table S1 is reporting the results of the included studies about non-respiratory-related outcome measures (for example: anxiety and depression).
