# Supplementary material for: The Effects of Digital Health Interventions for Pulmonary Rehabilitation in People with COPD: A Systematic Review of Randomized Controlled Trials
Source: Medicina (Kaunas). 2024 Jun 11;60(6):963. doi: 10.3390/medicina60060963 (PMC11206104; doi:10.3390/medicina60060963)
Supplement: Supplementary file 1 [file medicina-60-00963-s001.zip › medicina-3038716-supplementary.pdf]

Table S1: Summary of included trials that investigated the Effects of Digital Health Interventions for Pulmonary Rehabilitation in People with COPD: A Systematic Review of Randomized Controlled Trials

Author (year)	Location of the trial	Sample size	Age years (mean±SD)	Technology Utilized	Exercise prescription	Comparison group	Secondary findings
Nguyen et al., (2008)	USA	50<br>eDSMP: 26<br>fDSMP: 24<br>Stage of COPD: mild to severe	Total: (69.5±8.5)<br>M:F=22:17<br>fDSMP: (70.9±8.6)<br>M:F=11:9<br>eDSMP: (68.0±8.3)<br>M:F=11:8	Web-Based Application	**Duration for intervention and follow up (0, 3, 6 months).**<br>**F:** endurance 4x/week or strengthening 3x/week.<br>**I:** Light to moderate Borg scale.<br>**T:** 30 min/session<br>**T:** Endurance, strengthening exercise	Education, skills training, and ongoing support for dyspnoea self-management, including independent exercise.	- CRQ- HRQOL improved over time in both groups (P<0.001).<br>- SF-36 physical composite scores↑ for both groups (P=0.04).<br>- 10 participants reported acute exacerbations of COPD.<br>- Both groups advanced in their stage of readiness for exercise with up to 84%.
Stickland et al., (2011)	Canada	409<br>Standard PR: 262<br>Telehealth PR: 147	Standard PR: (69.5±9.7)<br>M:F=125:137<br>Telehealth PR: (69.2±8.6)<br>M:F=78:69	Video Conferencing	**Duration for intervention in both groups:** 8 weeks, **follow up** at 6 months.<br>**F:** 2x/ week for 8 weeks.<br>**I:** Intensity personalised based on patient symptoms and capacity.<br>**T:** 2h/session. Education session for 1h/session<br>**T:** aerobic, resistance, flexibility, and breathing exercise	**F:** 2x/week for 8 weeks.<br>I: Intensity personalised based on patient symptoms and capacity.<br>**T:** 2h/session. Education session for 1h/session<br>**T:** aerobic, resistance, flexibility, and breathing exercise	- No differences in program adherence for both groups
Tabak et al., (2014)	Netherlands	34<br>IG: 18<br>CG: 16<br>Stage of COPD: mild to	**IG:** (65.2±9.0)<br>M:F=8:6<br>**CG:** (67.9±5.7)<br>M:F=11:5	Smart-phone Application (App)	**Both groups received usual care (medication/physiotherapy) for 4 weeks.**<br>**IG:**<br>**F:** 4 days/week	Usual care (medication/physiotherapy)	- Activity levels not affected by the intervention (P= 0.482).<br>- Both groups motivated to start any activity, IG (P=0.073), CG (P=0.023).

		very severe			**T,T:** Activity couch walking till 22.00 h.<br>**CG**: usual care		
Bourne et al., (2017)	UK	90<br>Online PR: 64<br>Face-to-face PR: 26<br>Stage of COPD: mild to severe	Online PR: (69.1±7.9)<br>M:F=41:23<br>Face-to-face PR: (71.4±8.6)<br>M:F=18:8	PR Programme (myPR)	**- Duration time:** 6 weeks both groups<br>**- Online PR (myPR):**<br>**F:** 2- 5x/week<br>**I:** Borg score measurement.<br>**T:** each week the duration of exercise increased by 30s, starting from 60s in week 1, to 3½ min in week 6.<br>**T:** 10 exercises (biceps curls, squats, push ups against wall, leg extensions in sitting position, upright row with weights, sit-to-stand, arm swings with a stick, leg kicks to the side, arm punches with weights and step-ups)	**Face to face PR:**<br>**F:** 2 supervised sessions and 3 at home per week.<br>**T:** 10 exercises carried out by myPR	- CAT score difference in the ITT was −1.0 in favour of online PR.<br>- HADS mean differences for ITT was −0.74 in favour of online PR.
Tsai et al., (2017)	Australia	37<br>TeleG: 20<br>CG: 17<br>Stage of COPD: mild to severe	CG: (75±9)<br>M:F=6:11<br>TeleG: (73±8)<br>M:F=12:7	Videocon-ferencing	**Duration of intervention:** 8 weeks for both.<br>**TeleG:**<br>**F:** 3/week<br>**I:** 60-80% Peak cycle work rate OR 80% of 6MWT speed (walking training).<br>**T:** Lower limb cycle ergometry, walking training and strengthening exercise<br>**T:** 15-30 min	**CG:** Usual medical management including optimal pharmacological intervention.	- SWA not significantly different between both groups (P=0.64).<br>- FBI-SF not significantly different between both groups (P=0.55).<br>- CAT not significantly different between both groups (P=0.06).<br>- HADS improved more in TG compared to CG in anxiety (P=0.04) and depression (P=0.001).<br>- PRAISE improved more in TG compared to CG (P=0.007)

Vasilopoulou et al., (2017)	Greece	150<br>Group A: 50<br>Group B: 50<br>Group C: 50<br>Stage of COPD: moderate to very severe	Group A: (66.9±9.6) M:F=44:3<br>Group B: (66.7±7.3) M:F=38:12<br>Group C: (64.0±8.0) M:F=37:13	Telephone, video conference, tablet	**Duration of intervention and follow up:** (2, 12 months).<br>**Group A:**<br>**F:** 144 sessions over 12 months<br>**I:** Each patient with a resource executes the exercise (depends in patient status).<br>**T:** Arm and leg exercise<br>**Group B:** Participants visited the hospital 2/week for 12 months.<br>**F:** 96 sessions performed over 12 months	**Group C**: optimal pharmacotherapy oxygen therapy, vaccination, regular follow-up	- Group A lower rate of ED visit compared to group B and group C (P<0.001).<br>- Both group A and B improved time spent in light and moderate daily physical activities over 12 months of follow-up more compared to group C.<br>- The compliance of group A over 12 months of follow-up was 93.5%
Wang et al ., (2017)	China	130<br>CG: 68<br>IG: 62<br>Stage of COPD: moderate to very severe	CG: (71.9±8.1) M:F=36:29<br>IG: (69.3±7.8) M:F=21:34	Web-Based Coaching Program	All participants received usual care before discharge. Duration: 12 months<br>**EHR:** Health education, and pulmonary rehabilitation instructions<br>**Pulmonary rehabilitation:**<br>**T:** Abdominal contraction, lip breathing, respiratory muscle exercise, aerobic exercise	Usual care before discharge.	

Godtfredsen et al., (2020)	Denmark	134 patients<br>PR: 67<br>PTR: 67<br>Stage of COPD: severe	(68.3±9.0)<br>55% women	Online	**Duration of intervention and follow up:** 10 weeks, with 3 and 12 months follow up.<br>**PTR Group**:<br>**F**: 10 weeks supervised on-line PTR with a screen at homes.	**PR Group:**<br>**F**: 10 weeks of conventional PR at the local site. Both groups performed:<br>**T:** Endurance, resistance, breathing techniques and nutritional support.<br>**T:** 10 weeks.	- No significant between group differences or changes in the secondary outcomes<br>- 30 STS slightly better with PR group after 12 months of follow up.<br>- PAL↓ with and ↑depression domain in HADS with PR group.
Hansen et al., (2020)	Denmark	134<br>PTR: 67<br>PR: 67<br>Stage of COPD: Moderate to severe	(68.3±9.0)<br>M:F=60:74<br>PTR: (68.4±8.7)<br>M:F=32:35<br>PR: (68.2±9.4)<br>M:F=28:39	Video-conference software system	**Duration of intervention:** 10 weeks.<br>**Follow up:** 22 weeks.<br>**PTR:**<br>**F:** 3/week for 10 weeks<br>**T:** 35 min<br>**T:** Warm-up, high repetitive time-based muscle endurance training, and patient education session	**CPR:**<br>**F:** 2/week (1 hospital, for 10 weeks)<br>**T:** 60 min.<br>**T:** Warm-up, endurance, resistance training, cool-down, and patient education sessions	- HADS scores improved in anxiety and depression with PTR compared to PR after intervention.<br>- EQ-5D no group exceeded the MCID.<br>- 30-s STS no group exceeded the MCID.<br>- CCQ not significantly different between groups after intervention and the follow up.<br>- PAL↓ in number of daily steps per day in the PR from the baseline to the end of the intervention and to 22 weeks of follow up. In PTR remained unchanged.
Galdiz et al., (2021)	Spain	94<br>CG: 48<br>IG: 46<br>Stage of COPD: moderate to very severe	CG: (63.0±6.6)<br>M 68.8; F 31.2<br>IG: (62.3±8.2)<br>M 65.2; F 34.8	Mobile phone app	**Duration of intervention:** 8 weeks<br>**Follow up:** 3, 9, 12 months.<br>**Intensive PR:**<br>**F:** 3/week<br>**T:** 1 h<br>**T:** Weightlifting, leg cycle ergometry, educational sessions	**CG:** Usual care<br>**F:** Every day<br>**T:** At least 1 hour<br>**T:** Walking, general educational material	- HRQoL improved with IG group after 12 months follow up in SF36-PCS (P=0.039) and SF36-MCS (P=0.001), but not in CG. CG have less P value scores.<br>- CRQ dimensions not significant changed after 12 months follow up. CRQ-E improved with IG (P=0.002), but not CG

							- Rates of adherence 92.4% in the IG and 84.4% in the CG
Bahadori et al., (2023)	Iran	IG: 38<br>CG: 38	IG: (44.1±14.1)<br>F:M=25:10<br>CG: (47.7±13.8)<br>F:M=29:6	Android application	**Duration of intervention:** 6 weeks<br>**IG:**<br>**F:** all day/6 weeks<br>**T:** PR education<br>**CG:**<br>**F:** 2/week<br>**T:** PR education<br>**T:** 30–60 min	**CG:** wore the activity tracker every day and used a smartphone for the assessments but no access to the app	- QoL score after PR education improved with IG (P<0.001) but not in CG (P=0.829)
Spiel-manns et al., (2023)	Switzerland	67<br>IG: 33<br>CG: 34<br>Stage of COPD: moderate to very severe	Total: (64.3±7.7)<br>M:F=34:33<br>IG: (66.1±6.8)<br>M:F=17:16<br>CG: (62.7±8.2)<br>M:F=17:17	Smart phone application	**Duration of Intervention:** 3 months.<br>**Follow up:** 6 months.<br>**IG:**<br>**F:** Daily<br>**I:** Progressive increase based on patient feedback.<br>**T:** 15–20 min<br>**T:** Warm up, strength and mobility, stretching exercises.		- PA significant differences between both groups after 6 months of follow up (P=0.007). IG improved more than CG.<br>- Number of steps after 6 months of follow up higher in IG compared to CG (P=0.014).<br>- PA and number of steps not significant between both groups from the baseline to 3 months follow up (P≥0.199).<br>- HADS not significant changed in IG and ↑ in CG (P=0.033) and total score not significant after 6 months of follow up in either group (P=1.00).<br>- STST improved in IG compared with CG (P=0.004) after 3 months follow up and no significant difference between both groups (P=0.143) after 6 months follow up<br>- Sleep duration and efficiency not significantly changed in either group at 3 months for IG and CG, respectively (P=0.704), (P=0.294) and 6 months follow up (P=0.048), (P=0.120).

Zanabni et al., (2023)	Norway	120<br>TeleRG: 40<br>UC: 40<br>CG: 40<br>Stage of COPD: moderate to very severe	TeleRG: (64.9±6 7.1)<br>M:F=23:17<br>UC: (64.0±7.7)<br>M:F=20:20<br>CG: (63.5±8.0)<br>M:F=23:17	Videoconferencing sessions	**Duration of intervention:** 6 months.<br>**Follow up:** 1 and 2 years.<br>**TeleRG:**<br>**F:** 3-5/week<br>**I:** Moderate to high intensity Borg scale<br>**T:** Treadmill and strength exercises<br>**T:** At least 30 min<br>**UG:**<br>**T:** Treadmill exercises only as prescribed for Tele PR group	**CG:**<br>Standard care	- Incidence of hospitalization and ED lower in TeleRG (P=0.0008) and UC (P=0.0002) compared to CG<br>- PGIC improved in TeleRG compared to UG and CG (P=0.001) after 6 months of follow up<br>- GSES and HADS no significant difference between groups.<br>- No treadmill-related injuries were reported during the study
All the included trials were Randomized controlled trials: eDSMP: Internet-based Dyspnoea Self-management Program; fDSMP: face-to-face Dyspnoea Self-Management Program; 6MWT: 6-Minute Walk Test; CRQ: Chronic Respiratory Questionnaire; ADL: Activities of Daily Living; HRQOL: Health-Related Quality of Life; SF-36: Medical Outcomes Study Short-Form 36; PR: Pulmonary Rehabilitation; SGRQ: St George's Respiratory Questionnaire; 12 MWT: 12 Minute Walk Test; ESWT: Endurance Shuttle Walk Test; MRC: Medical Research Council Dyspnoea Scale; IG: Intervention Group; CG: Control Group; CCQ: Clinical COPD Questionnaire; MFI-20: Multidimensional Fatigue Inventory; 6MWD: 6 Minute Walk Distance; SNAPPS: Smoking, Nutrition, Alcohol consumption, Physical activity, Psychosocial well-being, and Symptom management; ISWT: Incremental Shuttle Walk Test; SWA: Sense Wear Armband; FBI-SF: Functional Performance Inventory–Short Form; TUG: Timed Up and Go; HADS: Hospital Anxiety and Depression Scale; PRAISE: The Pulmonary Rehabilitation Adapted Index of Self Efficacy; ED: Emergency Department; HER: Electronic Health Records; FEV1: Forced Expiratory Volume in the first second; FVC%: Percent of Forced Vital Capacity; PEF: Peak Expiratory Flow; MMEF: Maximal Mid Expiratory Flow; mMRC: modified Medical Research Council; PTR: Pulmonary Tele-Rehabilitation; STS: Sit to Stand; EQ-5D: EuroQol 5- Dimension Questionnaire; QoL: Quality of Life; PAL: Physical Activity Level; HR: Heart Rate; SOBQ: Shortness of Breath Questionnaire; CB: Caregiver Burden; STST: Sit To Stand Test; PA: Physical Activity; UC: Unsupervised Group; GSES: Generalized Self-Efficacy Scale; PGIC: Patient Global Impression of Change.							
